# A novel polymorphism (901G > a) of C5L2 gene is associated with coronary artery disease in Chinese Han and Uyghur population

**DOI:** 10.1186/1476-511X-12-139

**Published:** 2013-09-28

**Authors:** Ying-Ying Zheng, Xiang Xie, Yi-Tong Ma, Yi-Ning Yang, Zhen-Yan Fu, Xiao-Mei Li, Xiang Ma, Bang-Dang Chen, Fen Liu

**Affiliations:** 1 Department of Cardiology, First Affiliated Hospital of Xinjiang Medical University, Urumqi 830054, P.R. China; 2 Xinjiang Key Laboratory of Cardiovascular Disease Research, Urumqi 830054, P.R. China

**Keywords:** Acylation stimulating protein, C5L2, Triglyceride synthesis, Coronary artery disease

## Abstract

**Background:**

C5L2, a G protein-coupled receptor (GPCR), has been demonstrated to be a ligand for acylation-stimulating protein (ASP). The aim of the present study is to evaluate the association of a novel variation (901A > G) of C5L2 gene with coronary artery disease (CAD).

**Methods:**

We identified a novel single nucleotide polymorphism (SNP), (901G > A), in exon 2 using a polymerase chain reaction direct-sequencing method. This nucleotide change causes the amino-acid order from Arginine to glutaminate at codon 300. We analyzed the relationship between this SNP and CAD in two independent case–control studies: one was in a Han population (492 CAD patients and 577 control subjects) and the other was in a Uygur population (319 CAD patients and 554 control subjects).

**Results:**

The frequency of AG genotype in CAD subjects was less than that in the control subjects not only in Han (1.8% vs 8.6%, P < 0.001, OR = 0.143, 95% CI: 0.068 ~ 0.302) but also in Uygur population (0.9% vs 5.2%, P = 0.001, OR = 0.246, 95% CI: 0.072 ~ 0.837). After adjustment for known CAD risk factors such as hypertension, diabetes, smoking, age and gender, the difference remained significant.

**Conclusion:**

The 901G > A polymorphism of C5L2 may be a genetic maker of CAD in the Han and Uygur population in western China.

## Background

Coronary artery disease (CAD) is a complex multifactorial disorder involved multiple environmental risks and genetic factors [1,2] which causes most of death in many countries, including China. Genetic factors have been defined as important risk contributors to the pathogenesis of CAD [3-7]. More importantly, in China, the prevalence of obesity and type 2 diabetes mellitus (T2DM), which is important risk factor for atherosclerosis and CAD [8-10], is increasing rapidly. In recent years, most studies have focused on the associations between polymorphic variants in candidate genes and the risk of developing CAD [11-13].

In humans, Acylation Stimulating Protein (ASP) levels are increased in obesity, type 2 diabetes (T2DM), and cardiovascular disease. ASP is a human plasma protein that stimulates both triacylglycerol synthesis and glucose transport via its receptor C5L2. C5L2 is a potential ligand for both C5a (a powerful inflammatory factor) and ASP. It added one addition association between the adipose and the immune system. Other study have demonstrated that ASP binds to C5L2, initiating a cascade of events that includes phosphorylation, β-arrestin translocation, and receptor internalization. Activation of C5L2 initiates protein kinase C activation and translocation and glucose transporter translocation [14-16]. This resulting in a net accumulation of adipose TG stores [11,12,17] and leading to an increase concentration in obesity.

In a previous study [13], we identified a novel SNP (698C > T) in C5L2 gene which causes an amino acid change from Proline to Leucine at codon 233. We have demonstrated that (698C > T) is associated with CAD and T2DM in Chinese Han and Uygur population. In this study we aimed to examine the relationship between another novel SNP (901G > A) and CAD in a Chinese Han and a Uygur population.

## Results and discussion

Table 1 shows the clinical characteristics of CAD patients and control subjects. In Han subjects, the following variables were significantly different between the two groups: diabetes; smoking; drinking; the serum concentration of glucose; TC; HDL-C; LDL-C and creatinine (all P < 0.05). There was no significant difference in the following variables between CAD patients and control subjects: Hypertation; serum concentration of TG; BUN; the body mass index (BMI); age; and sex (all P > 0.05). In Uygur subjects, the following variables were also significantly different between these two groups: hypertension; diabetes; smoking; drinking and the serum concentration of glucose, HDL-C, and TG (all P < 0.05). There was no significant difference in the following variables between CAD patients and control subjects: the serum concentration of TC; LDL-C, creatinine and BUN; the BMI; age and sex (all P > 0.05).

**Table 1:** Characteristics of participants

		**Han**				**Uygur**		
	**Control (n = 577)**	**CAD ** **(n = 492)**	**χ**^**2 **^**or *t***	***P *value**	**Control (n = 554)**	**CAD ** **(n = 319)**	**χ**^**2 **^**or *t***	***P *value**
Age, mean (SD)	57.70 (11.75)	58.45 (10.29)	1.591	0.112	49.89 (17.78)	49.69 (14.47)	-.181	0.856
Sex, female (%)	129 (0.224)	104 (0.211)	1.015	0.314	90 (19.12)	62 (15.38)	1.43	0.138
Hypertension, n (%)	309 (53.5)	315 (64.0)	10.979	0.001	141 (26.26)	116 (38.67)	13.93	<0.001
Diabetes, n (%)	167 (28.9)	241 (49.0)	44.876	<0.001	63 (11.87)	83 (27.67)	32.91	<0.001
Smoking, n (%)	225 (39.0)	291 (59.1)	41.884	<0.001	210 (38.11)	168 (53.16)	17.96	<0.001
Drinking, n (%)	184 (31.9)	184 (37.4)	3.394	0.065	158 (28.52)	116 (36.36)	5.78	0.01
BMI, mean (SD)	25.67 (3.30)	184 (37.4)	1.563	0.118	26.37 (4.04)	26.84 (4.86)	-1.48	0.786
Glucose, mean (SD)	4.88 (0.97)	6.22 (2.34)	-12.378	<0.001	5.51 (1.82)	5.93 (2.40)	-2.72	<0.001
TG, mean (SD)	1.85 (1.66)	2.03 (1.82)	-1.600	0.110	1.92 (1.82)	1.84 (0.90)	0.727	<0.001
TC, mean (SD)	4.49 (1.03)	4.17 (1.03)	4.805	<0.001	4.53 (1.23)	4.21 (1.04)	3.50	0.235
HDL-C, mean (SD)	1.33 (0.40)	1.13 (0.32)	8.406	<0.001	1.17 (0.63)	1.00 (0.28)	4.14	<0.001
LDL-C, mean (SD)	2.93 (1.02)	2.52 (0.85)	6.819	<0.001	2.68 (0.94)	2.62 (0.93)	0.874	0.869
UA, mean (SD)	330.64 (91.68)	331.47 (85.94)	-0.149	0.881	304.75 (87.04)	321.99 (87.91)	-2.62	0.009
Cr, mean (SD)	74.25 (18.37)	78.53 (26.50)	-3.039	0.002	78.41 (22.81)	78.70 (21.81)	-1.71	0.120
BUN, mean (SD)	4.99 (1.50)	5.29 (1.72)	-3.010	0.003	5.16 (1.74)	5.23 (1.79)	-0.521	0.602

The genotype distribution of 901A > G did not show a significant difference from the Hardy–Weinberg equilibrium values in both ethnicities (Both P > 0.05 in CAD group and control group). The frequency of the heterozygote carriers of the 901 GA genotype of C5L2 was significantly lower in CAD patients than that in control subjects not only in Han (1.8% versus 8.6%; P < 0.001) but also in Uygur (5.2% versus 0.9%; P =0.001) (Table 2). The frequency of A allele in CAD patients was also lower than that in control subjects both in Han (1% Versus 4%, P < 0.001) and in Uygur subjects (3.0% versus 1.0%; P = 0.001) (Table 2). The odds ratio (OR) for carriers of the 901GA genotype for CAD was 0.205 [95% confidence interval (CI): 0.100–0.423] in Han subjects and 0.172 [95% CI: 0.052–0.569] in the Uygur population. After adjustment of confounders such as hypertension, diabetes, smoking, systolic blood pressure, diastolic blood pressure, and the serum concentration of HDL-C, LDL-C, creatinine and BUN, the difference remained significant not only in Han subjects (P <0.001, OR = 0.143, 95% CI: 0.068–0.302) but also in Uygur population (P < 0.001, OR = 0.246, 95% CI: 0.072–0.837) (Table 3).

**Table 2:** Distribution of genotypes and alleles of C5L2 gene

	**Group**	**n**	**Genotype (n, %)**		***P***	**Allele (Frequency)**		***P***
			**GG**	**AG**		**G**	**A**	
Han population	Control	577	529 (98.3)	48 (8.6)	<0.001	0.96	0.04	<0.001
	CAD	492	483 (92.7)	9 (1.8)		0.99	0.01	
Uygur population	Control	554	525 (94.8)	29 (5.2)	0.001	0.97	0.03	0.001
	CAD	319	316 (99.1)	3 (0.9)		0.99	0.01	

**Table 3:** Results of logistic regression

	**Uighur**						**Han**					
	**B**	**S.E.**	**χ2**	**P**	**OR**	**95% C.I.**	**B**	**S.E.**	**χ2**	**P**	**OR**	**95% C.I.**
901G > A	-1.401	0.624	5.037	<0.001	0.246	0.072 ~ 0.837	-1.944	0.381	25.955	<0.001	0.143	0.068 ~ 0.302
Smoking	0.590	0.158	10.431	0.001	1.663	1.221 ~ 2.265	0.964	0.130	54.925	<0.001	2.621	2.031 ~ 3.382
HDL-C	-1.394	0.284	24.050	<0.001	0.248	0.142 ~ 0.433	-0.533	0.196	7.360	0.007	0.587	0.400 ~ 0.863
Constant	2.097	0.711	8.173	<0.001	8.145		2.194	0.466	22.158	<0.001	8.971	

In the present study, we identified a novel SNP (901G > A) and found that the GA genotype is associated with decreased risk for CAD in a Han and in a Uygur population of Xinjiang, the western China.There are increasing evidences of close interactions between immune systems and adipose biology, which is a concept now well recognized within metabolic research in the areas of obesity, insulin resistance, diabetes and cardiovascular disease, as highlighted by numerous review articles [18-20]. C5L2, a G protein coupled receptor (GPCR), is a recently identified receptor for C5a, C5adesArg, as well as ASP/C3adesArg and C3a [18,20]. In the present study, we identified a novel SNP (901G > A) and assessed the association between this SNP and CAD in a Han population and in a Uygur population. We found the frequency of the GA genotype was significantly lower in CAD patients than in control subjects. Logistic regression analyses suggested that, after adjustment for other cardiovascular risk factors, the GA genotype remained associated with decreased risk for CAD not only in Han population (OR = 0.143 P < 0.001, 95% CI = 0.068 ~ 0.302), but also in Uygur population (OR = 0.264 P < 0.001, 95% CI = 0.072 ~ 0.837).

In the previous study, Michel et al. [21] identified a novel variant (S323-to-I substitution) in the C5L2 gene which was associated with familial combined hyperlipidemia in a French–Canadian family. Gain-of-function studies in human C5L2 stably transfected HEK-293 (HEK-hC5L2) cells [22] showed that TG synthesis and glucose transport were significantly increased upon ASP stimulation compared with non-transfected cells, resulting in net accumulation of adipose TG stores and insulin sensitivity. These data suggested that C5L2 was associated with hyperlipidemia and diabetes, which have been reported to be risk factors of CAD. And we also found that HDL-C level was lower in CAD patients than in control subjects in the Uygur population (*P* <0.05). Hence, we also examined if the relationship between the C5L2 genetic variant and CAD is modified by the concentration of HDL-C and diabetes. We found the relation between 901G > A variant and CAD was not modified by other factors including diabetes.

There were several limitations in the present study. Firstly, the present study was limited by the relatively small sample size. This may have led to weak statistical significance and wide CIs when estimating ORs. Secondly, we did not examine the expression of C5L2 between CAD and control group. Finally, we did not analyze the difference between each genotype in 901G > A polymorphism.

In conclusion, CAD was associated with the GA genotype of 901G > A in the human C5L2 gene. This result may broaden the knowledge of genetic variants and disease-association studies. Undertaking genome-wide association studies in different populations certainly merits investigation.

## Subjects and methods

Two case–control studies (Han and Uygur) were studied independently. Subjects diagnosed with CAD at the First Affiliated Hospital of Xinjiang Medical University from January 2006 to December 2009 were recruited. In this hospital approximately 2500 patients undergo coronary angiography every year and we selected 492 Han patients and 319 Uygur patients for this study. CAD diagnosed by angiography, which was defined as the presence of at least one significant coronary artery stenosis of ≥50% luminal diameter on coronary angiography. To ensure matching for age and gender, we selected 554 Han and 577 Uygur healthy subjects from the Cardiovascular Risk Survey (CRS) study which was described previously [23-25]. Briefly, the CRS consists of 14,618 subjects (5 757 Hans, 4 767Uygurs, and 4 094 Kazakhs). These individuals did not have: a history of CAD; electrocardiographic signs of CAD. Demographic data information about the presence of traditional coronary risk factors, including hypertension, diabetes mellitus, smoking, and serum cholesterol, were collected from all study participants.

Hypertension was defined as having a systolic blood pressure above140 mmHg or/and diastolic blood pressure above 90 mmHg or any anti-hypertensive agent used.

Dyslipidemia was diagnosed according the current guidelines from the National Cholesterol Education Program (NCEP) Adult Treatment Panel (ATP) III, that any abnormal status of TG, HDL-C and LDL-C (TG ≥ 1.70 mmol/L, HDL-C < 0.91 mmol/L, LDL-C ≥ 3.46 mmol/L), Hypercholesterolaemia was defined as a documented total cholesterol value ≥200 mg/dl (≥5.2 mmol/L) [26], or current treatment with cholesterol lowering treatment. Diabetes was determined by abnormal fasting plasma glucose levels [27] or the current use of insulin or an oral hypoglycemic medication. Body mass index (BMI) was calculated as body weight (kg) divided by height squared (m^2^) in units of kg/m^2^. All participants underwent a standardized physical examination performed by experienced research staff. Standing body height (cm), weight (kg) and waist circumference (WC, cm) were noted previously [23-25]. Informed consent was obtained from each individual according to a protocol approved by the Ethics Committee of the First Affiliated Hospital of Xinjiang Medical University.

### Primer design and C5L2 gene sequencing

Genomic DNA extraction from peripheral blood samples and complete sequencing of the C5L2 region that included a portion of the upstream region, 1 intron, and the exons have been described previously [28]. Sequencing reactions were undertaken by BGI-Beijing (Beijing, China; http://www.genomics.cn), sequencing primers were designed using Primer Premier 5.0 software. The sense primer was 5’ TGCTCACTG TGGCGGCCCCGA3’ and the antisense primer was 5’CCTTTTTAGGCACTAGAG ATA3’.

### Genotyping of novel SNPs

**Figure 1:**
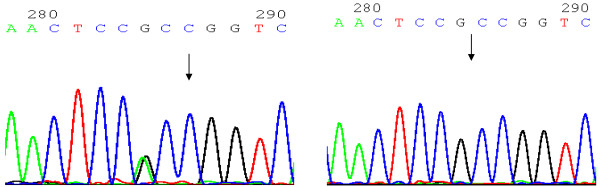
The sequencing of C5L2 gene G901A polymorphism, mutation was marked with arrows, (Left) GA genotype; (Right) GG genotype).

The detailed of genotyping of novel SNP have been previously described. Briefly, DNA sequencing of complete C5L2 region using a polymerase chain reaction direct-sequencing method, we identified a heterozygous variant (901G > A) in 48 subjects with CAD (Figure 1) resulting the amino-acid order substitution from Arginine to glutaminate at codon 300 in the exon 2. Genotyping for the 901G > A in the present case–control study was done by PCR amplification of 166 bp in exon 2 followed by restriction digestion with Age I (Fermentas, Beijing, China). The sense primer was 5’ 5’CTCAATCCCATGCTCTTCC3’ and the antisense primer was 5’CTCCAGCCTA CACCTC CA3’.

### Statistical analyses

Statistical analysis was performed using the SPSS version 17.0 software (SPSS, Chicago, IL, USA),Data are expressed as the mean ± standard deviation (SD). The significance of differences was evaluated using the t-test for continuous variables and the χ^2^ test for non-continuous variables. The differences between CAD patients and control subjects were assessed by independent-sample t-test. Categorical variables such as allele and genotype frequencies among CAD cases and controls were compared by using the Chi-square test. Hardy-Weinberg equilibrium was assessed by chi-square analysis. Multivariate analysis was performed using a logistic regression analysis for independent variables that were related to the presence or absence of CAD. A value of P < 0.05 was considered significant.

## Abbreviations

SNP: Single nucleotide polymorphisms; CAD: Coronary artery disease; ASP: Acylation-stimulating protein; TG: Triglycerides; TC: Total cholesterol; HDL-C: High-density lipoprotein; LDL-C: Low-density lipoprotein.

## Competing interests

The authors declare that they have no competing interests.

## Authors’ contributions

YYZ and XX carried out the molecular genetic studies and drafted the manuscript.YNY, ZYF and XML carried out the genotyping. XM and BDC participated in the design of the study and performed the statistical analysis. YTM, FL and YYZ conceived of the study and participated in its design and coordination and helped to draft the manuscript. All authors read and approved the final manuscript.
